# Surface modification of biodegradable magnesium and its alloys for biomedical applications

**DOI:** 10.1093/rb/rbu013

**Published:** 2014-11-28

**Authors:** Peng Tian, Xuanyong Liu

**Affiliations:** State Key Laboratory of High Performance Ceramics and Superfine Microstructure, Shanghai Institute of Ceramics, Chinese Academy of Sciences, Shanghai 200050, PR China

**Keywords:** magnesium alloys, surface modification, coatings, ion implantation, biodegradability, biocompatibility

## Abstract

Magnesium and its alloys are being paid much attention recently as temporary implants, such as orthopedic implants and cardiovascular stents. However, the rapid degradation of them in physiological environment is a major obstacle preventing their wide applications to date, which will result in rapid mechanical integrity loss or even collapse of magnesium-based implants before injured tissues heal. Moreover, rapid degradation of the magnesium-based implants will also cause some adverse effects to their surrounding environment, such as local gas cavity around the implant, local alkalization and magnesium ion enrichment, which will reduce the integration between implant and tissue. So, in order to obtain better performance of magnesium-based implants in clinical trials, special alloy designs and surface modifications are prerequisite. Actually, when a magnesium-based implant is inserted *in vivo*, corrosion firstly happens at the implant-tissue interface and the biological response to implant is also determined by the interaction at this interface. So the surface properties, such as corrosion resistance, hemocompatibility and cytocompatibility of the implant, are critical for their *in vivo* performance. Compared with alloy designs, surface modification is less costly, flexible to construct multi-functional surface and can prevent addition of toxic alloying elements. In this review, we would like to summarize the current investigations of surface modifications of magnesium and its alloys for biomedical application. The advantages/disadvantages of different surface modification methods are also discussed as a suggestion for their utilization.

## Introduction

Metallic biomaterials have played an important role in implant applications in loading bearing conditions, such as orthopedic implants and cardiovascular stents, where their high mechanical strength and fracture toughness make them to be superior to ceramics, polymers and polymer/ceramic composites. The early metallic materials used as biomedical implants consist of stainless steel [1, 2], Co–Cr-based alloys [3–6], titanium-based alloys [6–12], zirconium-based alloys [13, 14] and tantalum-based alloys [15, 16]. All these kinds of biomedical metals cannot degrade *in vivo* and will permanently exist in human body after implantation. The adverse effects including an increased risk of local inflammation may be caused by corrosion debris [17, 18] or toxic elements released into surrounding environment [19] in long-term existence of these implants. So improving their corrosion resistance by alloying or surface modifications is prerequisite to improve their surface stability and prevent toxic ions from releasing. Because of existence of these potential dangers, a second surgery is usually conducted for implant removal after the injured tissue healing. For cardiovascular stent application, the biodegradability of the inserted stents is more meaningful because if the previous intervention treatment fails or symptoms relapse, a new stent can be implanted into the same site [20, 21]. Furthermore, the mechanical properties of these traditional non-degradable metals are not in complete accord to those of natural tissues and the mechanical mismatch will result in some adverse effects. The mechanical mismatch between bone tissue and these metallic implants will result in a clinical phenomenon known as stress shielding. As we all know, bone tissue is constantly undergoing remodeling and modification in response to imposed stresses produced by normal everyday activities. The stress shielding usually leads the surrounding bone tissue to experience a reduced loading stress, which ultimately leads to bone resorption [22].

Biodegradable non-metal materials, such as polymers [23, 24], ceramics [25] or bioactive glasses [26], have been widely investigated as substitute of these non-degradable metallic implants. These biodegradable materials perform better on preventing the adverse effects caused by long-term existence of the non-degradable implants as they can gradually degrade *in vivo* and non-toxic degradation products are absorbed or excreted by surrounding tissue. However, these materials have a common disadvantage in load-bearing applications according to their innate mechanical properties. Considering a proper mechanical strength, biodegradable metals are promising candidates in load-bearing situations, where a high mechanical strength and a suitable Young’s modulus are required [27]. As trace elements existing in the human body, magnesium [28], iron [29] and newly investigated zinc [30]-based materials have been investigated as promising candidates for temporary implant materials. Among these kinds of biodegradable metals, magnesium and its alloys have been widely and systematically investigated for biomedical applications.

Magnesium is a lightweight metal with a density of around 1.74 g/cm^3^, which is slightly less than that of natural bone which ranges from 1.8 to 2.1 g/cm^3^. The elastic modulus of pure magnesium is 45 GPa and human bone varies between 40 and 57 GPa. Because of this similarity in elastic modulus, using magnesium in hard tissue engineering applications would greatly reduce the possibility of stress shielding, thus, preventing bone resorption. So considering the match of mechanical properties, magnesium-based materials are the best choice for biodegradable orthopedic implants. Some typical mechanical properties of tissues and biomaterials are summarized in Table 1.

**Table 1:** Typical mechanical properties of tissues and biomaterials

Tissue/material	Density (g/cm^3^)	Compressive strength (MPa)	Tensile strength (MPa)	Yield strength (MPa)	Elastic modules (GPa)	Elongation (%)
Arterial wall			0.50–1.72		0.001	
Collagen			60		1.0	
Collagen (rat tail tendon)					3.75–11.5	
Cancellous bone	1.0–1.4	1.5–9.3	1.5–38		0.01–1.57	
Cortical bone	1.8–2.0	160 Trans.	35 Trans.		5–23	
		240 Long.	283 Long.			
Cobalt–chrome alloys	7.8		450–960		195–230	
Stainless steel	7.9		480–620		193–200	
Titanium alloys	4.4		550–985		100–125	
Synthetic hydroxyapatite	3.05–3.15	100–900	40–200		70–120	
Alumina ceramics	3.30–3.99	2000–4000			260–410	
(Al_2_O_3_ 80–90%)						
Polymethylmethacrylate	1.12–1.20	45–107	38–80		1.8–3.3	
(PMMA)						
Polyethylene-	1.31–1.38	65–90	42–80		2.2–3.5	
terephthalate (PET)						
Pure magnesium	1.74	20–115	90–190		45	
AZ31 (Extruded)	1.78	83–97	241–260		45	
AZ91D (Die cast)	1.81	160	230		45	
Mg–6Zn		433.7 ± 1.4	279.5 ± 2.3	169.5 ± 3.6	42.3 ± 0.1	18.8 ± 0.8
Mg–1Ca (cast)			71.38 ± 3.01			1.87 ± 0.14
Mg–1Ca (rolled)			166.7 ± 3.01			3 ± 0.78
Mg–1Ca (extruded)			239.63 ± 7.21			10.63 ± 0.64
Mg–0.6Ca		273.2 ± 6.1		114.4 ± 15.1	46.5 ± 0.6	
Mg–1.2Ca		254.1 ± 7.9		96.5 ± 6.6	49.6 ± 0.9	
Mg–1.6Ca		252.5 ± 3.3		93.7 ± 7.8	54.7 ± 2.4	
Mg–2.0Ca		232.9 ± 3.7		73.1 ± 3.4	58.8 ± 1.2	
Mg–2Sr (rolled)			213.3 ± 17.2	147.3 ± 13.1		3.15 ± 0.3
Mg–6Ag		244.1 ± 9.2	215.9 ± 11.3		45 ± 1	
Mg–0.5Ca–0.5Sr		274.3 ± 7.2				
Mg–1.0Ca–0.5Sr		274.2 ± 4.0				
Mg–0.1Ca–1.0Sr		214.5 ± 3.5				
Mg–1Zn–1Mn (cast)			174	44		12
Mg–1Zn–1Mn (extruded)			280	246		22

*Note*: Data compiled from Refs [28, 51, 52, 54, 55, 57, 60, 61].

In addition to possessing the proper mechanical strength, magnesium and its alloys are chosen as promising candidate for biodegradable implants because of their biocompatibility and safety *in vivo*. As fourth most abundant cations in human body, with approximately half of the total magnesium stored in bone tissue, magnesium is essential to human metabolism [31]. Magnesium is a cofactor for many enzymes, and acts as a stabilizer for the structures of DNA and RNA [32]. As bivalent ion, it takes part in apatite formation in bone matrix and is also used in a number of metabolic processes in human body [33]. The normal level of magnesium concentration in extracellular fluid ranges between 0.7 and 1.05 mmol/l, where homeostasis is maintained by the adjustment of kidney and intestine. So the incidence of hyper-magnesium is rare due to the efficient excretion of excess magnesium ions through urine [34]. So the degradation of magnesium-based implants in human body generally will not cause an increased level of serum magnesium [35]. As we all know, dissolved ions from metal implants are always a concern to induce allergy. However, the previous report shows that magnesium alloys, such as AZ31, AZ91, WE43 and LAE442, will not cause allergenic reactions in an epicutaneous patch test in accordance with ISO standard [36]. Moreover, the major corrosion product Mg(OH)_2_ has been proved to be closely related to the enhanced bone formation and temporarily decreased bone resorption [37].

Magnesium and its alloys are generally considered to degrade in physiological environment via a corrosion process, which produces a corrosion product layer mainly composed of magnesium hydroxide and simultaneously release hydrogen gas. The corrosion process happens basing on an electrochemical reaction. The overall corrosion reaction of magnesium in aqueous solution can be given as below:

1 $${\text{Mg}}_{\left(\text{s}\right)}+2{\text{ H}}_{2}{\text{O}}_{\left(\text{aq}\right)}⇌\text{Mg}{\left(\text{OH}\right)}_{2\left(\text{s}\right)}+{\text{H}}_{2(\text{g})}\;$$

This overall reaction may be divided into the following partial reactions:

2 $${\text{Mg}}_{\left(\text{s}\right)}⇌{\text{Mg}}_{\left(\text{aq}\right)}^{2+}+2{\text{ e}}^{-}\;\left(\text{anodic reaction}\right)$$

3 $$2{\text{ H}}_{2}{\text{O}}_{\left(\text{aq}\right)}+2{\text{e}}^{-}⇌{\text{H}}_{2(\text{g})}+2{\text{ OH}}_{\left(\text{aq}\right)}^{-}\;\left(\text{cathodic reaction}\right)$$

4 $${\text{Mg}}_{\left(\text{aq}\right)}^{2+}+2{\text{ OH}}_{\left(\text{aq}\right)}^{-}⇌\text{Mg}{\left(\text{OH}\right)}_{2(\text{S})}\;\left(\text{production formation}\right)$$

Although the magnesium hydroxide layer accumulated on the underlying magnesium substrate during the corrosion process can act as a protective layer preventing the following corrosion, when the chloride concentration in surrounding environment rises above 30 mmol/l, the magnesium hydroxide is prone to convert into magnesium chloride, which is highly soluble in water. Therefore, the spontaneously formed magnesium hydroxide layer cannot protect the substrate effectively. Severe corrosion and rapid degradation are inevitable for magnesium-based implant *in vivo* where the chloride content is about 150 mmol/l [38], which is much higher than that magnesium hydroxide can bare.

After implantation, magnesium-based implants undergo severe corrosive attack *in vivo*. Although for magnesium-based implants, the released metallic ions are considered to be physiologically beneficial since these ions can be consumed or absorbed by the surrounding tissues, or be dissolved and readily excreted by kidney [34], the rapid degradation of magnesium-based implants not only affects their long-term mechanical integrity *in vivo*, but also causes some adverse effects to human body. In orthopedic and cardiovascular stent applications, implants are usually required to undertake a certain load during the healing of injured bone tissues or vascular walls [28, 39]. Although the biodegradable implants are allowed to degrade finally, the implants still need to possess enough mechanical strength during their service time. With rapid degradation, the mechanical strength and integrity of the magnesium-based implants will be deteriorated seriously. In the late period of their service, the implants nearly lose their all load-bearing ability. Pits or cracks caused by rapid corrosion may result in sudden catastrophic and premature cracking causing eventually failure of the implants, even at the initial stage after implantation [40, 41]. Local alkalization and magnesium ion enrichment are the two common phenomena occurred around the magnesium-based implants according to their rapid corrosion, which produces much more OH^−^ anions and Mg^2+^ cations than that tissues can absorb or transport. In most *in vitro* experiments, the pH value and magnesium ion concentration of the immersion solution are significantly increased by magnesium corrosion [42]. Due to the efficient excretion of the excess magnesium in the urine by kidney and pH value adjustment by body fluid, the degradation of magnesium-based implants *in vivo* usually may not cause any problem. However, the disequilibrium of microenvironment around the implant will affect the attachment and proliferation of cells, thus retarding the tissue healing process. To be specially pointed out, the patients, who suffer from diseases in kidney, should be cautious about the choice of magnesium-based implant. The large number of magnesium ions resulting from the degradation of magnesium-based implant will increase the burden of kidney, which may deteriorate the disease. The inefficient of magnesium excretion will result in an increase of serum magnesium level, which may cause many dangerous complications [43]. The rapid degradation of magnesium implant *in vivo* also leads to an accumulation of hydrogen nearby the implant because of limited hydrogen gas adsorption ability of the tissues [27, 44]. Gas cavity is usually considered to be unfavorable for the integration of the implants and tissues. However, although most patients experienced subcutaneous gas cavities by rapid degradation of the implants, they had no pain and almost no infections were observed during the postoperative following up in the early clinical cases [45]. When an implant is inserted into human body, a contact with blood is usually inevitable, especially when used as cardiovascular stent. Low hemolysis ratio is critical for blood contacting materials considering the minimum harm to blood cells, which is required to be under safety value of 5% according to ISO 10993-4. Magnesium and its alloys with no treatment usually cause severe hemolysis due to their rapid degradation [46]. As discussed in the field of biodegradable materials, a two-way relationship between the material and the biological host response has been proposed, i.e. the degradation process or the corrosion products can induce local inflammation and the products of inflammation can enhance the degradation process [47, 48]. Although the definite mechanism of the relationship between corrosion process and local inflammation is generally unknown for biodegradable metals, some previous results have shown that fast corroding magnesium alloys respond with a mild foreign body reaction [49, 50].

In order to control the degradation rate of magnesium-based implants to maintain their mechanical strength as well as reduce the side effects mentioned above during their service time, many new magnesium alloys have been designed especially for biomedical applications by adding alloying elements. Mg–Ca [51, 52], Mg–Zn [53, 54], Mg–Sr [55] and Mg–Ag [56, 57] alloys have been developed by adding single nutrient element or antibacterial element. Mg–Zn–Zr [58], Mg–Zn–Ag [59], Mg–Ca–Sr [60], Mg–Zn–Mn [61] and Mg–Nb–Zn–Zr [62, 63], etc. complex alloys have also been developed for better performance in mechanical properties and biocompatibility. Compared with commercial magnesium alloys, such as AZ31 alloy and WE43 alloy, although these newly developed magnesium alloys possess better mechanical properties, corrosion resistance and biological performance, alloying alone does not reach the requirement of corrosion resistance due to the high electronegative potential of magnesium (−2.4 V with respect to hydrogen electrode) and its poor passivating tendency. Moreover, the inhomogeneous microstructures in magnesium alloys may cause localized corrosion, which will cause accelerated following corrosion process. So it is critical to minimize the localized corrosion of magnesium-based implants, especially during the initial stage of post-implantation, to maintain enough strength to support injured tissues with minimum side effects. Moreover, the response of surrounding tissues to the implants is closely related to their surface properties [64]. So for magnesium-based implants, proper surface corrosion resistance and good biocompatibility for surrounding tissues integration with the implants are critical for their applications.

Surface modification of magnesium and its alloys is aimed to construct an anti-corrosion layer with good surface biocompatibility. After surface modification, the mechanical properties of bulk substrate usually maintain. Moreover, surface modification is feasible to construct a multifunctional surface on magnesium-based implants for better biomedical performance. So it is of great importance to review the current developments of surface modification methods for magnesium-based implants. Advantages and disadvantages of each method are also discussed to give a suggestion for their usage in different situations.

## Surface Modification of Magnesium and Its Alloys for Biomedical Applications

**Figure 1.**
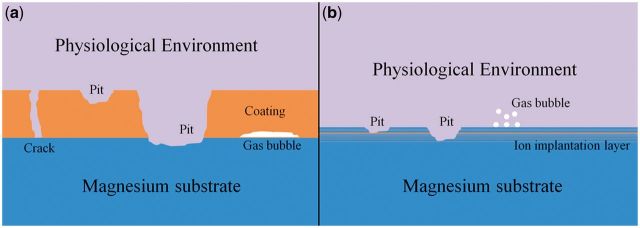
Schematic diagram illustrating the corrosion failure mechanism of surface modified magnesium and its alloys: (**a**) coated magnesium substrate and (**b**) ion implanted magnesium substrate.

For biodegradable magnesium-based implants, the aim of the surface modification is just to control their degradation rate and improve their surface biocompatibility, but not permanently change the surface structures and properties, such as leading to non-degradability of implants or toxicity to surrounding tissues. Hence, a biodegradable dynamic interface should be considered in order to endow the implants with desirable corrosion resistance and surface biocompatibility as well as maintain the mechanical strength of the substrate during service stage [65]. Considering the existence of interfaces and failure mechanism of the modification layers, coatings, or called films and ion implantation can be divided into two main classes. Figure 1 depicts the schematic diagram of the failure mechanism of the coated (a) and ion implanted (b) magnesium substrate during corrosion process.

### Coatings on magnesium and its alloys

Coatings are promising to minimize the initial localized corrosion of magnesium and its alloys. Usually being a layer with a proper thickness, the coatings on magnesium-based implants can undertake the role for protecting the substrate from severe corrosion, especially at the initial stage after implantation. Moreover, as a temporary surface, the proper designed coating can gradually disappear *in vivo* and will not produce deleterious effects to the surrounding tissues. According to whether the magnesium substrate involved or not in the coating formation, three classes can be divided to substrate involving coatings, non-substrate involving coatings and composite coatings. The current progresses of various coatings formation are reviewed as follows and Pros and Cons of different coatings are also discussed.

#### Substrate involving coatings

In fact, the substrate involving coatings are products through the reaction of magnesium substrate and treatment reagent. As the coatings form *in situ*, they usually adhere highly to magnesium substrate, which is important for reducing the potential danger that the whole or partial coating may be delaminated. However, because the magnesium substrate is involved in the coating formation, the formed coatings mainly consist of magnesium compounds, which are not satisfactory for cells attachment and proliferation presenting poor surface biocompatibility.

##### Chemical conversion coatings

Chemical conversion treatment is a sufficient method to form coatings on magnesium and its alloys to control their corrosion rate. During the treatment process, the whole or partial contents of treatment solution can react with the magnesium substrate to form magnesium compounds, which constitute a protective layer retarding the subsequent corrosion of magnesium substrate.

**Figure 2.**
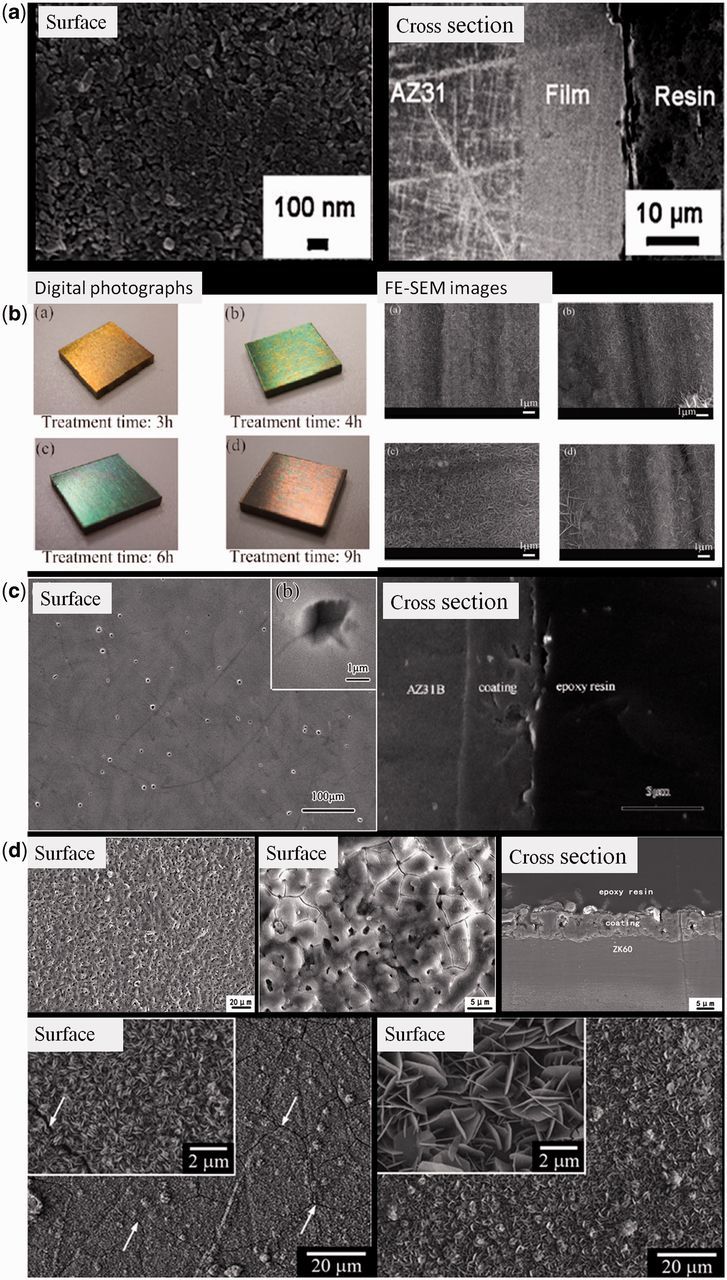
(**a**) Surface and cross-sectional morphologies of Mg(OH)_2_ film by NaOH treatment for 3 h [66]. (**b**) Digital photographs and FE-SEM images of color-tuned surfaces on AZ31 alloy by water treatment [69]. (**c**) Surface and cross-sectional morphologies of fluoride-treated AZ31 alloy for 72 h. The insert shows the high magnification [71]. (**d**) Surface and cross-sectional morphologies of the MAO-coated Mg–Zn–Zr alloy [76]. (**e**) Surface morphologies of Mg–Fe–CO_3_ LDH layers on pure magnesium by different treatment process [80].

Alkaline treatment can be used to modify magnesium-based materials because Mg(OH)_2_ can be formed as a mainly protective layer. With treatment by 5.66 wt.% NaOH solution at 160°C, Zhu *et al*. [66] fabricated a Mg(OH)_2_ film on AZ31 alloy (Fig. 2a). By the protection of Mg(OH)_2_ film, the corrosion rate of the magnesium alloy was inhibited effectively. During corrosion in Hank’s solution, amorphous calcium apatite precursor was observed to deposit on the surface of the film. The tape test revealed a strong adhesion between the film and the substrate. In cytotoxicity tests, no signs of changes on cell morphology or inhibitory effect on cell growth were detected for this kind of film [67]. Apart from alkaline solution containing NaOH, three weak alkaline solutions (Na_2_HPO_4_, Na_2_CO_3_ and NaHCO_3_) were also applied for Mg–Ca alloy treatment [68]. After soaked in these solutions and subsequently heat treated, the corrosion rates of Mg–Ca alloy in simulated body fluid were effectively decreased with the following sequence: NaHCO_3_ heated < Na_2_HPO_3_ heated < Na_2_CO_3_ heated. Moreover, cytotoxicity evaluation showed that none of the alkaline heat-treated Mg–Ca alloy samples induced toxicity to cells. Without any chemical additions, Ishizaki *et al*. [69] only used ultrapure water to fabricate Mg(OH)_2_ coating on AZ31 alloy. Vertically self-aligned nano- and microsheets with color expression were formed at a temperature of 120°C with different treatment time (Fig. 2b). The color-tuned magnesium alloy showed anti-corrosive performance and damping capacity. With further modification with *n*-octadecyltrimethoxysilane, color-tuned superhydrophobic surfaces were successfully formed. Because the coatings formed by alkaline treatment mainly consists of Mg(OH)_2_, which is easily attacked by chloride ions to convert into highly water soluble MgCl_2_, the coatings formed by this method may not reach the required corrosion resistance in long-term service in chloride-rich physiological environment.

Compared with Mg(OH)_2_, MgF_2_ is a much stable phase in physiological environment. Hydrofluoric acid immersion is commonly used to fabricate MgF_2_ coating on magnesium alloys by surface fluoridation (Fig. 2c). Carboneras *et al*. compared the effect of HF treatment of powder metallurgy Mg, cast Mg and AZ31 alloy. The formed MgF_2_ coating on their surface slowed down the degradation rate of all the alloys, especially for cast Mg and AZ31, retarding the corrosion process in cell culture medium for at least a week [70]. During the degradation process, the fluoride-treated magnesium alloy maintained better mechanical strength evaluated by three-point bending test, presenting a promise for application as biodegradable implants [71]. In *in vivo* test, a new bone formed at the edges of the MgF_2_-coated magnesium implants and the degradation induced a calcium and phosphorous rich layer on the implant surface, which was covered by an incomplete layer containing fluoride. The MgF_2_-coated implant showed a slight decrease in volume and better strength after 6 months implantation compared with uncoated implants [72]. MgF_2_ coating achieves a big success in modification of magnesium-based implants by *in vitro* and *in vivo* evaluations for biomedical applications. However, the hydrofluoric acid is commonly used in MgF_2_ formation, which may be dangerous for operators and harmful to environment. So fluoride salt may be a promising substitute and proper treatment parameters need to be investigated.

Surface phosphorylation is considered to improve the corrosion resistance as well as surface bioactivity of magnesium-based materials to form a phosphate-containing coating. Ye *et al*. [73] used phytic acid (PA) to fabricate a conversion coating on WE43 alloy by simple immersion treatment. Their work showed that the PA treatment could enhance the corrosion resistance of the magnesium substrate and the cytocompatibility of the PA-coated WE43 alloy was significantly improved. Moreover, the hemolysis ratio of PA-coated WE43 alloy was lower than 5%, which met the hemolysis standard of biodegradable materials. Considering that phosphorous is a kind of nutrient element, especially for bone growth in human being, surface phosphorylation of magnesium-based materials is more promising for orthopedic application.

Apart from the conversion coatings mentioned above, rare earth conversion treatment on the surface of magnesium alloys is an environmentally friendly technology. Moreover, a small content of rare earth element does not influence human health based on the practice application of the rear earth containing magnesium alloys mentioned above. Cui *et al*. [74] investigated the corrosion behavior of cerium conversion coating on AZ31 magnesium alloy in physiological solution. The formed dense Ce-based conversion coating consisted of a mass of trivalent and tetravalent cerium oxides. The coating could provide obvious protection for magnesium substrate to effectively reduce the degradation speed in Hank’s solution. Unlike the traditional method, Levy *et al. *[75] developed a diffusion coating of Nd on Mg-1.2%Nb-0.5%Y-0.5%Zr-0.4%Ca alloy and investigated the effect of this coating on corrosion behavior of the alloy in simulated physiological electrolyte. By the protection of the diffusion coating, the corrosion resistance of the alloy was significantly improved. The obtained enhancement of corrosion resistance was due to the formation of a relatively continuous network of passive Mg_41_Nd_5_ intermetallic at grain boundaries and the enrichment of the oxide film with Nd and Nd-oxide. Rare earth conversion coatings are mainly composed of the corresponding compounds. As these compounds present better corrosion resistance, they can effectively separate the magnesium substrate from corrosive fluids. Although the rare earth elements used have not been reported to present obvious toxicity, the potential danger of these elements in long term should be paid attention.

##### Plasma electrolytic oxidation coating

Plasma electrolytic oxidation (PEO), also called micro-arc oxidation (MAO), has been widely applied to fabricate porous and robust coatings on biodegradable magnesium and its alloys, which has been widely used in the improvement of corrosion resistance of magnesium-based implants. Yang *et al*. [76] used this technology to fabricate a Mg_2_SiO_4_ containing coating on ZK60 magnesium alloy (Fig. 2d). The MAO coating not only significantly enhanced the corrosion resistance of magnesium alloy, but also improve its *in vitro* biocompatibility. The extract of MAO-coated alloy showed no cytotoxicity and lead to an increase of alkaline phosphatase level compared with that of naked alloy, indicating that the release of Mg and Si ions from the coating was beneficial for the differentiation of bone marrow stromal cell (BMSCs). For cells directly grown on various surfaces, the surface of MAO coating exhibited a better cell adhesion and affinity. The hemolysis ratio of MAO-coated alloy (1.04%) was drastically decreased compared with that of the naked alloy (28.89%), meaning a great improvement of the hemocompatibility. By simply adjusting the compositions of electrolytes, Ca–P containing coating [77, 78] and ZrO_2 _containing coating [79] were also successfully fabricated. Compared with other methods, PEO may be more promising to fabricate coatings on magnesium-based materials because the formed coatings are hard and highly adhered to the substrate. Moreover, as mentioned above, the compositions of the coatings are easily adjusted by simply adjusting the compositions of electrolytes, which is promising in biomedical applications because nutrient elements or antibacterial elements can be introduced into the coatings. However, because of the existence of surface pores and some cracks formed during PEO process, the PEO coatings are still not satisfactory in anti-corrosion in long-term service as corrosive fluids can penetrate into the holes and cracks, thus, significantly reducing the corrosion resistance of the coating. Moreover, the main phase composition of PEO coating is MgO, which is not suitable for cells growth. So the surface biocompatibility of this kind of coating is needed to be improved. So the PEO coating is required to be further modified to obtain enhance corrosion resistance as well as surface biocompatibility. So the PEO-based composite coatings are promising in practice and the progresses will be also reviewed in the following part.

###### Magnesium-based layered double hydroxide

Magnesium-based layered double hydroxide (LDH) is also introduced into surface modification of magnesium and its alloys (Fig. 2e). Lin *et al.* [80] fabricated highly oriented Mg–Fe–CO_3_ LDH coatings directly on pure Mg by a two-step treatment, i.e. treatment in pH 5.6 aqueous Fe^3+^/HCO^−^/CO_3_^2−^ at 50°C and then immersing it in pH 9.5 aqueous HCO^−^/CO_3_^2−^ at 50°C. The former step was performed to yield Mg^2+^ in aqueous solution by corroding the Mg substrate. A two-layered thin film was thus formed on Mg substrate with a outer layer comprised fine plate-like Mg–Fe–CO_3_ LDH. After the latter treatment in pH 9.5 aqueous HCO^−^/CO_3_^2−^ at 50°C, the fine LDH platelets grown into a strong-oriented Mg–Fe–CO_3_ LDH. The Mg–Fe–CO_3_ LDH-coated sample had a much higher corrosion resistance than the pure Mg substrate. Moreover, the LDH coating showed a better cell spreading and cell–cell interaction behavior than the pure Mg substrate. Because the composition of Mg containing LDH is restricted by selection of trivalent metal cations and anions, magnesium-based LDH is recently developed on surface modification of magnesium-based materials. Although researches about magnesium-based LDHs are rarely reported until now, the application of this method is promising as the compositions of LDHs have potential to be adjusted by the exchange of anions, which is beneficial for developing bio-functional surfaces.

#### Non-substrate involving coatings

Compared with substrate involving coatings, the non-substrate involving coatings are usually composed of other materials with proper corrosion resistance as well as biocompatibility. Inorganic coatings mainly composed of Ca–P compounds and organic coatings mainly composed of biodegradable polymers have been widely investigated.

##### Inorganic coatings

Inorganic coatings have been applied to improve the corrosion resistance of magnesium and its alloys, such as DLC coatings [81, 82], TiO_2_ coatings [83] and ZrN/Zr bilayered coating [84]. Although these coatings exhibit good corrosion resistance, the non-degradability limits their application in biomedical area, where the eventually degradation of the coatings is required. So only biodegradable inorganic coatings applied on magnesium and its alloys are summarized in this review.

Bioglasses have been widely used as bone cement and scaffold because of their excellent biocompatibility. Taking advantage of their excellent biocompatibility, bioglasses were coated on magnesium sponges. Biocompatibility and degradation behavior evaluation showed that all coated magnesium sponges were tolerated well and no gas evolution or severe bone alterations were observed. After implantation, different sized implant reduction and newly formed bone around the implant were observed [85].

Ca–P-based coatings are the most commonly used and investigated as biodegradable inorganic coatings to improve the corrosion resistance of magnesium and its alloys as well as their surface bioactivity. Many methods have been investigated to fabricate Ca–P-based coatings on magnesium and its alloys [86].

**Figure 3.**
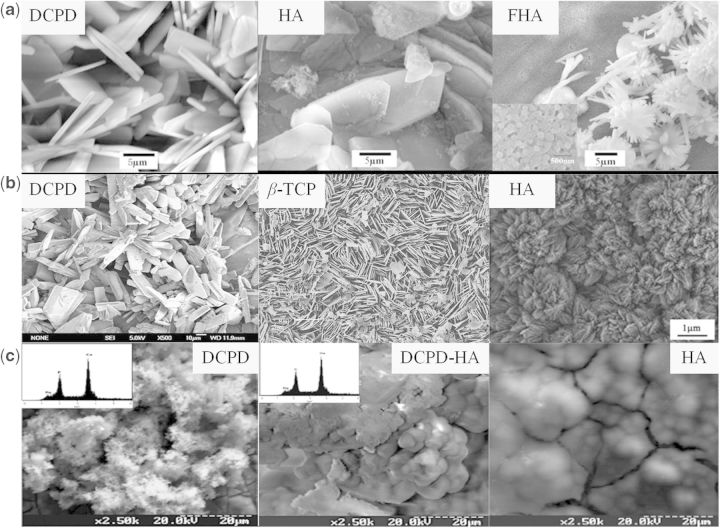
(**a**) Surface morphologies of DCPD, HA and FHA on Mg–Zn alloy by electrodeposition process [87]. (**b**) Surface morphologies of DCPD [92], β-TCP [94] and HA [96] on magnesium substrate by chemical solution treatment. (**c**) Surface morphologies of DCPD, DCPD-HA and HA on magnesium substrate by biomimetic process [98].

Electrodeposition is commonly used to deposit Ca–P-based coatings on magnesium and its alloys. Song *et al*. [87] used this method to fabricate brushite (DCPD, CaHPO_4_·2H_2_O), hydroxyapatite (HA, Ca_10_(PO_4_)_6_(OH)_2_) and fluoridated hydroxyapatite (FHA, Ca_5_(PO_4_)_3_(OH)_1−_*_x_*F*_x_* coatings on Mg–Zn alloy (Fig. 3a) and compared their corrosion behavior in modified simulated biological fluid. All of these coatings decreased the degradation rate of Mg–Zn alloy. The precipitates on the uncoated and DCPD-coated Mg–Zn alloy in modified simulated biological fluid had low Ca/P molar ratio, which delayed bone-like apatite formation, while both HA and FHA coating could promote the nucleation of osteoconductive minerals for 1 month. However, the HA coating transformed from DCPD through alkali heat treatment was fragile and less stable, and therefore its long-term corrosion resistance was not satisfactory. Instead, the FHA was more stable and had better corrosion resistance. Li *et al*. [88] found that the bone-like FHA also showed better cellular proliferation and differentiation than Mg–Zn substrate for human BMSCs (hBMSCs).

Compared with traditional cathodic electrodeposition process, a modified pulse electrodeposition has also been applied to fabricate Ca–P-based coatings. Wang *et al*. [89] reported the deposition of soluble Ca-deficient hydroxyapatite (Ca-def HA) coating on Mg–Zn–Ca alloy substrate by pulse electrodeposition. By regulating the pulse amplitude and width to appropriate parameters, the Ca-def HA coating showed better adhesion to Mg–Zn–Ca alloy. With this coating protection, the corrosion resistance was significantly improved, thus the ultimate tensile strength and time of fracture for the coated Mg–Zn–Ca alloy were higher than those of the untreated one. They also evaluated this coating *in vivo*. The valid life of the coating is about 8 weeks, after that the degradation rate of the coated implants increases obviously. Histopathological examination showed that the Ca-def HA coating had good osteoconductivity and was in favor of the formation of more new bone on the surface of magnesium alloy [90]. By adding H_2_O_2_ in preparation electrolyte, a dense and uniform nano fluorine-doped hydroxyapatite (HA) coating was prepared by this method. The coating could effectively protect Mg substrate from corrosion. Moreover, compared with traditional cathodic electrodeposition coating, the pulse electrodeposition coating could more effectively induce the precipitation of Mg^2+^, Ca^2+^ and PO_4_^3−^ [91].

Chemical solution containing Ca and P contents has also been used to directly fabricate Ca–P-based coatings on magnesium and its alloys by simply chemical treatment (Fig. 3b). DCPD coating composed of bar-shaped crystals was deposited on the surface of magnesium by chemical treatment in an aqueous solution containing Na_2_HPO_4_ and Ca(NO_3_)_2_. The DCPD coating showed protection of the substrate at the initial corrosion stage [92]. Xu *et al*. [93] studied the porous and netlike DCPD layer formed on the surface of the Mg alloy. *In vitro* cell evaluation showed that L929 cells presented significantly good adherence and proliferation on the Ca–P-coated magnesium alloy. *In vivo* evaluation results demonstrated that the Ca–P coating provided magnesium with a significantly good surface bioactivity and promoted early bone growth at the implant/bone interface. Pure β-tricalcium phosphate (β-TCP) was also investigated by this method [94]. Tomozawa *et al*. [95] formed HA coatings uniformly on pure Mg by a hydrothermal treatment using a C_10_H_12_N_2_O_8_Na_2_Ca (Ca-EDTA) solution. This HA coating remarkably reduced the corrosion rate of the Mg in simulated body fluid (SBF). Moreover, the biological responses, including cell attachment, proliferation and differentiation, of the HA-coated samples were enhanced considerably compared with the pure magnesium. Preliminary *in vivo* experiments showed that the biodegradation of Mg implant was significantly retarded by this HA coating [96].

As we all know, the hydroxyapatite is formed *in vivo* by the regulation of collagen. The hydroxyapatite is formed spontaneously in the mild biological environment, which is called biomineralization. Ca–P-based materials deposition in simulated biological fluid by the inducement of materials surface composition and structure is called biomimetic process. Zhang *et al*. formed a homogenous bone-like apatite coating successfully on pure magnesium using a biomimetic method without the need of heat treatment. The corrosion rate of the magnesium implants could be closely tailored by adjusting the apatite coating thickness [97]. In biomimetic process, variation of preparation parameters could lead to a big difference in finally formed coating. For example, the variation of ionic composition of the initial solution led to the deposition of coatings with various phase composition, i.e. DCPD, DCPD + HA, HA (Fig. 3c) and the magnetic field influenced the particle morphology and crystal texture of the precipitates [98].

Apart from these commonly used methods, cold spray process [99] and transonic particle acceleration process [100] have also been used to fabricated Ca–P-based coatings on magnesium and its alloys.

##### Organic coatings

**Figure 4.**
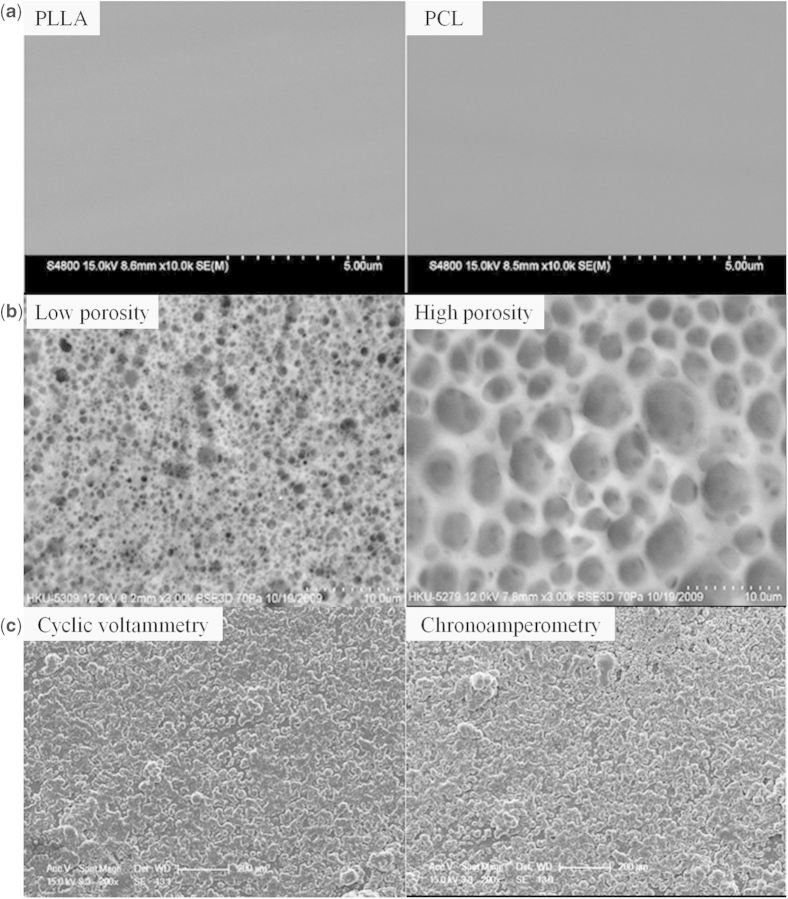
(**a**) Surface morphologies of compact polymer coatings on magnesium substrate [101]. (**b**) Surface morphologies of porous PCL coatings on AZ91 alloy [35]. (**c**) Surface morphologies of PEDOT conductive coatings fabricated on magnesium with different processes [104].

Degradable polymers have been applied in various areas including biomedical applications [23, 24]. As possessing good biocompatibility, these polymers are good choice as coatings for magnesium and its alloys. Among those polymers, polylactide (PLA) and polycaprolactone (PCL) are mostly investigated. Xu and Yamamoto [101] prepared biodegradable polymer films of PLLA and PCL on magnesium by spin coating in order to improve its early corrosion resistance and cytocompatibility. The amorphous PLLA and semi-crystalline PCL coatings presented uniform and nonporous surface structure on Mg (Fig. 4a). The adhesion strength evaluation showed that PLLA film had better adhesion strength to Mg substrate than that of PCL film and for both PLLA and PCL films, low molecular weight film was thinner and exhibited better adhesion strength than high molecular weight one. With these polymer films protection, the corrosion resistance of Mg substrate was improved and the polymer surfaces were suitable for SaOS-2 cells attachment and growth. Apart from nonporous coatings, a pore size controllable PCL coating (Fig. 4b) was also fabricated by Wong *et al*. [35] on magnesium alloy. In addition to reduce the degradation rate, the bulk mechanical properties of magnesium substrate were also maintained during degradation process. Moreover, good *in vitro* cytocompatibility of eGFP and SaOS-2 osteoblasts was obtained by the polymer coating. The *in vivo* study indicated that the polymer coatings retarded the degradation of the implants and higher volumes of new bone were observed on the polymer-coated sample. Poly(lactic-co-glycolic acid) (PLGA) is artificially synthesized polymer which has good biocompatibility and controlled degradation rates with different ratio of PLA/PGA. Using PLGA polymer in solution at various concentrations, Ostrowski *et al*. [102] fabricated coatings of varying thickness on magnesium alloy substrates. Although the coatings initially provided protection for the substrate to reduce degradation over 3 days, they did not maintain a reduction in corrosion rate after this time point. Inhomogeneous coating durability and gas pocket formation during degradation resulted in eventual detachment from the alloy surface. *In vitro* studies of cell viability showed improved biocompatibility of polymer-coated substrates. So although long-term degradation control was not obtained, short protection and improved biocompatibility of magnesium alloys were achieved by the PLGA polymer coatings. As these polymers have been used as biodegradable implants, their safety and biocompatibility have been identified. Because the magnesium substrate plays a role as a supporter, the lack of mechanical strength of these biodegradable polymers is also prevented. However, because the polymers are just physically adhered to the magnesium substrate, presenting an obvious interface between magnesium substrate and the polymer coatings, the adhesion strength may not reach the requirement for biomedical applications, causing a potential danger of polymer coatings peeling off during the degradation.

Surface modification of magnesium-based implants by grafting biofunctional molecules is a feasible method to construct a functional surface with good biological performance.

Liu *et al*. [103] fabricated biofunctionalized anti-corrosive silane coatings on magnesium alloys using bistriethoxysilylethane and 3-amino-propyltrimethoxysilane. To obtain better corrosion resistance, the layer of densely crosslinked bistriethoxysilylethane coating was immobilized on NaOH-activated Mg surface. Then 3-amino-propyltrimethoxysilane was grafted onto the pretreated surface to impart amine functionality to the surface. Furthermore, heparin was covalently conjugated onto the silane-treated magnesium alloy to render the coating hemocompatibility, as indicated by reduced platelet adhesion on the heparinized surface. So by constructing multilayer on magnesium alloys step by step, a multifunctional surface can be easily obtained.

For neural applications, Sebaa *et al.* [104] used poly(3,4-ethylenedioxythiophene) (PEDOT) as a conductive coating to control the degradation and improve the cytocompatibility of magnesium substrate. The coatings showed porous structure and adhesion strength within the classifications of 3B to 4B (Fig. 4c). The corrosion resistance was significantly improved by the coatings. Moreover, the PEDOT coatings could load the anti-inflammatory drug dexamethasone during the electrodeposition, which could be subsequently released upon electric stimulation [105].

The surface modification by formation of self-assembling monolayers of nontoxic organic molecules is a facial way to design a functional surface for biomedical applications. Grubac *et al*. [106] fabricated alkylphosphonate self-assembled films on AZ91D alloy. The existence of well organized and ordered self-assembled alkylphosphonate monolayers showed good protecting properties in physiological solution. Ishizaki *et al*. [107] used vapor phase method to fabricate alkanoic and phosphonic acid-derived self-assembled monolayers on magnesium alloy. The contact angle hysteresis of SAMs with a carboxylate headgroup is much larger than that of SAMs with a phosphonic acid group. The phosphonic acid-derived SAMs had higher molecular density and better corrosion resistance compared with alkanoic acid-derived SAMs.

#### Composite coatings

Considering the requirement of multifunctional surface of magnesium and its alloys, mainly enough corrosion resistance and biofunctions, usually different kinds of coatings are combined to fabricate composite coatings on magnesium and its alloys. Taking advantages of different kinds of coatings, the composite coatings usually possess combined properties of enhanced corrosion resistance and biocompatibility.

**Figure 5.**
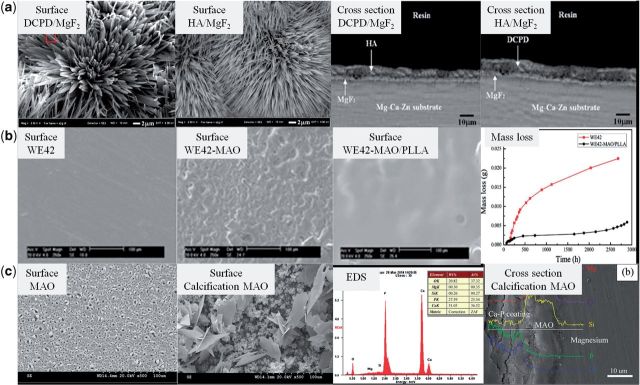
(**a**) Surface and sectional morphologies of DCPD/MgF2 and HA/MgF2 composite coatings on Mg–Ca–Zn alloy [108]. (**b**) Surface morphologies of WE42, WE42-MAO, WE42-MAO/PLLA and corrosion mass loss of WE42, WE42-MAO/PLLA [109]. (**c**) Surface morphologies and elemental compositions of MAO and calcification MAO coatings, cross-sectional morphology of calcification MAO coating [111].

Magnesium fluoride (MgF_2_) coating and hydroxyapatite coating have been widely applied on surface modification of magnesium and its alloys. Combining these two kinds of coatings, Bakhsheshi-Rad *et al*. synthesized nano-hydroxyapatite/magnesium fluoride (nano-HA/MgF_2_) coating and dicalcium phosphate dehydrate/magnesium fluoride (DCPD/MgF_2_) composite coating via fluoride conversion process followed by electrochemical deposition on magnesium alloy (Fig. 5a). The root mean square roughness of the nano-HA/MgF_2_ and DCPD/MgF_2_ composite coatings was approximately 395 and 468 nm, respectively, which is higher than that of fluoride treated and untreated samples. The needle-like HA crystals had a diameter of 80–150 nm and a length of about 7 μm and the plate-like DCPD was relatively larger. The composite coatings reduced the hydrogen evolution and improved the nucleation site of apatite compared with that of the uncoated sample [108].

Using the chemical conversion coatings as inner layers, Kunjukunju *et al*. fabricated multilayered coatings of alginate and poly-l-lysine on alkaline- and fluoride-pretreated magnesium substrate using a layer-by-layer technique. Furthermore, surface functionalization of these coatings by chemical crosslinking and fibronectin immobilization was conducted to control the cellular activity of these multilayered films [42]. Cytocompatibility studies showed that the fluoride conversion is more suitable as pretreatment method because better bioactivity and less cytotoxicity were obtained compared with the hydroxide pretreatment. Although imparting good biocompatibility of the modified surface, the multilayered coatings of alginate and poly-l-lysine did not alter the degradation kinetics of the substrates and it is the pretreatment conditions that had a significant impaction on the overall coating degradation behavior.

MAO coatings are proved to significantly improve the corrosion resistance of magnesium. However, the porous structure of MAO coatings limits their long-term protection for magnesium substrate. Also considering the inadequate biocompatibility, the MAO coatings may be not proper being used alone as modification surface of magnesium-based implants. Guo *et al*. [109] fabricated a composite MAO/poly-l-lactic acid (MAO/PLLA) coating on the surface of WE42 alloy (Fig. 5b). The PLLA coating effectively sealed the microcracks and micropores on the surface of MAO coating by physical interlocking to inhibit the severe attack of corrosive fluid. The corrosion rate was decreased and the cytocompatibility was improved by the MAO/PLLA composite coating protection. Moreover, the MAO/PLLA composite coating endowed magnesium substrate with good hemocompatibility [110]. Also based on MAO coating, Liu *et al*. [111] formed a calcium phosphate coating on its surface by chemical method to construct MAO/Ca–P composite coatings (Fig. 5c). The outer calcified coating was composed of calcium-deficient HA and DCPD. After SBF incubation, some new apatite formed on the calcified coating surface. Compared with PEO coating only, the composite coating increased the corrosion potential and decreased the hydrogen gas release to present better corrosion resistance.

Compared with single kind of coating, the composite coatings indeed take the advantages of combined coatings. Commonly, one coating plays a role as anti-corrosion layer and the other one plays a role as biofunctional layer. Considering the biomedical application, the composite coatings have more potential because of their enhanced multifunction. Although rare investigations have been reported, potential dangers of coating dropping off may be caused because more interfaces are introduced in formation of composite coatings.

### Ion implantation

Ion implantation involves a process in which ions are accelerated and impinge into the modified surface. This technique provides the possibility of introducing different species into a substrate independent of thermodynamic limitations such as solubility. Ion implantation introduces a suitable amount of ions into the near surface of the materials to alter the surface properties such as corrosion resistance and biocompatibility. Unlike surface coatings, an ion implanted layer does not have an abrupt interface and layer delamination may be not a serious issue [65]. Ion implanted surface is very thin, so it usually does not provide enough protection in long term. However, for retarding initial corrosion, ion implantation is feasible and effective. According to the implanted elements, ion implantation for magnesium modification can be classified as gas ion implantation, metal ion implantation and dual ion implantation.

#### Gas ion implantation

Gas ion implantation can introduce inorganic elements, such as oxygen and nitrogen, into magnesium substrate. Wan *et al*. [112] used oxygen plasma immersion ion implantation to control the degradation rate of magnesium substrate. Their study showed that although the treated sample presented enhanced corrosion resistance against neutral PBS, they could not withstand the more aggressive chloride ion enriched PBS. The enhanced corrosion resistance is considered to ascribe to increased Mg–O bonding states formed on the surface layer of magnesium and more homogenous surface morphology due to the ion bombardment effects. Because the Mg–O bonding can be dissolved easily in Cl^−^ enriched and more acidic ambience, the enhancement of corrosion resistance was reduced in chloride ion-enriched PBS. Tian *et al.* [113] conducted nitrogen plasma ion implantation on AZ31B alloy. The improvement of corrosion resistance after nitrogen implantation was considered to be attributed to the compactness of the loose natural oxide layer and ion irradiation effect. Severe surface sputtering and possible formation of a small amount of Mg_3_N_2_ phase might have an adverse effect.

Tian *et al.* [114] also applied water plasma ion implantation and oxidation for magnesium alloys to improve their corrosion resistance. The oxide layer consisted of a native oxide layer and oxidization layer induced by water implantation. With increasing treatment time and voltage, the oxygen content in the layers increased. The corrosion resistance could be effectively improved using the proper water implantation and oxidation conditions. The improved corrosion resistance was attributed to the formation of a compact oxide layer. Considering that the modified surface is mainly composed of magnesium oxide, water implanted surface may be also easily attacked by chloride ion rich environment.

**Figure 6.**
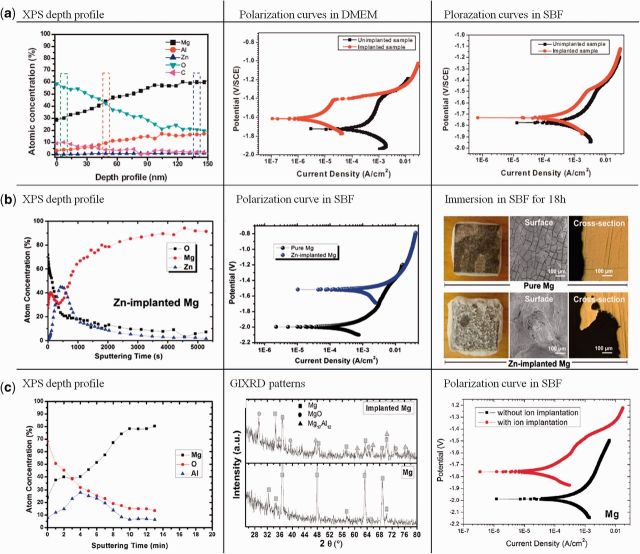
(**a**) XPS depth profile of CO_2_-PIII-treated AZ31 alloy and polarization curves of untreated and CO_2_-PIII-treated AZ31 alloy in DMEM and SBF [115]. (**b**) XPS depth profile, polarization curve in SBF and corrosion in SBF for 18 h of Zn-PIII-treated pure Mg [116]. (**c**) XPS depth profile, GIXRD pattern and polarization curve in SBF of Al-PIII-treated pure Mg [117].

Recently, Xu *et al*. [115] investigated the effects of carbon dioxide plasma immersion ion implantation on the electrochemical properties of AZ31 magnesium alloy in physiological environment. A surface layer with carbon in the graphite state and an oxide film composed of magnesium oxide and aluminum oxide was formed in the near surface of AZ31 by carbon dioxide PIII. The surface modification improved the corrosion resistance especially in Dulbecco's Modified Eagle Medium (DMEM) (Fig. 6a).

#### Metal ion implantation

Gas ion implantation improves the corrosion resistance of magnesium and its alloys mainly by passivating their surface to form an oxygen- or nitrogen-rich layer. With different mechanism, metal ion implantation can introduce metallic elements into the magnesium substrate to form surface alloying. In biomedical applications, the biological properties and toxicity of the alloying elements must be considered. As we all know, aluminum (Al) and zinc (Zn) are common used as alloying element in AZ-based alloys which have obtained a success in industry. However, after Zn implantation, the degradation rate in simulated body fluids was increased significantly. The X-ray photoelectron spectroscopy (XPS) results revealed that a thin Zn rich surface layer with Zn existing in the metallic state was formed by ion implantation, which attributed to the decrease of corrosion resistance due to the galvanic effect (Fig. 6b) [116]. In contrary with the results of Zn ion implantation, Al ion implantation appreciably improved the surface corrosion resistance of pure Mg as well as AZ31 and AZ91 alloys. This enhancement could be attributed to the formation of a gradient surface structure with a gradual transition from an Al-rich oxide layer to Al-rich metal layer revealed by XPS depth profile (Fig. 6c) [117]. However, considering the potential danger of Al, the Al ion implantation must be cautiously chosen for biomedical application.

Zirconium (Zr), titanium (Ti) and tantalum (Ta) are biologically friendly to the human body as their metal or oxide implants have been applied clinically. So Ti, Zr and Ta have been tried as implanted ions into magnesium and its alloys. Liu *et al.* [118] conducted Ti and Zr ion implantation for AZ91 magnesium alloy. The surface layers showed a characteristic intermixed layer consisting of a outer surface mainly composed of titanium or zirconium oxide with a small amount of MgO and Mg(OH)_2_, an intermediate layer containing metal oxide and metallic implanted particles, and a bottom layer rich in metallic elements. With the implantation of Ti and Zr ions, the corrosion resistance of AZ91 alloys was improved. Wang *et al*. [119] found that Ta ion implantation could also improve the corrosion resistance of AZ31 alloy. Ta_2_Al was found to be produced in the modified layer. The mechanism for improved corrosion resistance of the implanted samples could be ascribed to the formation of a pre-oxidation layer with a duplex structure of the dense MgO layer and the protective Ta_2_Al barrier.

#### Dual ion implantation

**Figure 7.**
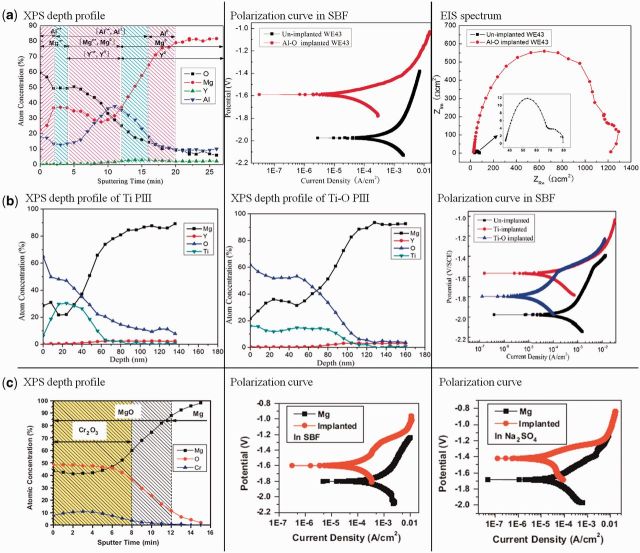
(**a**) XPS depth profile, polarization curve and EIS spectrum of Al–O dual ion implantation treated WE43 alloy [120]. (**b**) XPS depth profiles and polarization curves of Ti and Ti–O dual ion implantation treated WE43 alloy [122]. (**c**) XPS depth profile and polarization curves in SBF and Na_2_SO_4_ of Cr–O dual ion implantation treated pure Mg [125].

Generally, it is difficult to avoid oxidation when the samples are exposed to air or an oxygen-containing environment. Some results also shows that metal ion implantation can results in O-rich outer layer which may contribute to the improvement of corrosion resistance for magnesium substrate. Considering the better corrosion resistance of metal oxide, construction of an oxide layer on magnesium surface is feasible and can be fabricated by metal and oxygen dual implantation. Zhao *et al*. employed aluminum and oxygen dual ion implantation to modify the surface of magnesium alloy (Fig. 7a). The results indicated Al and O ion implantation produced an Al_2_O_3_-containing protection layer. The modified layer improved the corrosion resistance of the substrate and localized corrosion became the dominant corrosion mechanism instead of general corrosion [120]. Just because of the protection of the modified Al_2_O_3_ layer, the plasma-treated implant degraded more slowly and simultaneously stimulated bone formation *in vivo* in a minimal invasive way without causing post-operative complications [121]. Titanium and oxygen dual ion implantation produced a TiO_2_-containing film which also significantly enhanced the corrosion resistance of magnesium alloy (Fig. 7b) [122]. ZrO_2_-containing surface film was fabricated on magnesium alloy by zirconium and oxygen dual ion implantation. Corrosion resistance, *in vitro* biocompatibility and even antimicrobial properties were enhanced [123]. Xu *et al*. also produced a thicker surface oxidized layer composed of chromium oxide by chromium and oxygen dual ion implantation. The formed layer could successfully retarded the surface degradation of pure magnesium [124]. In simulated body fluid and sodium sulfate, the chromium and oxygen dual ion implanted magnesium both had a lower corrosion rate and exhibited less pitting corrosion (Fig. 7c) [125].

## Concluding Remarks

Surface modification plays an important role in practical applications of magnesium-based materials evaluated by *in vitro* and *in vivo* tests, especially at the initial degradation stage of their service. By surface modification, better corrosion resistance and surface biocompatibility of magnesium-based materials are achieved.

Biodegradable coatings and ion implantation are widely investigated in surface modification of magnesium-based materials. Although these surface modification methods achieve a big success in improving the biomedical performance of magnesium-based materials, they have not formed a perfect surface on magnesium-based materials presenting good corrosion resistance and biocompatibility. Some works still need to be done:

1. Among substrate involving coatings, PEO coating is more promising considering its high adhesion strength to substrate and enhanced corrosion resistance. However, the long-term corrosion resistance of this coating is not satisfactory because of its surface porous structure. So reducing the porosity of the coating by adjusting preparation parameters or following sealing process is needed to be further investigated for practical application. Moreover, the PEO coating is usually lack of surface biocompatibility for cell attachment and proliferation on its surface, so more efforts should be done to improve its surface biocompatibility.
2. For non-substrate involving coatings, either inorganic coatings or organic coatings, a potential danger may happen when the coatings drop from the substrate wholly or partially because of their lack of adhesion strength. So for this kind of coatings, much more attention need to be paid in improving the adhesion strength of the coatings to magnesium substrate.
3. For ion implantation, the modified layer is very thin. So the enhancement of corrosion resistance for magnesium-based materials is not satisfactory for long term. However, ion implantation with proper choice of implanted ions can retard the initial corrosion of the magnesium substrate. When the magnesium substrate has better corrosion resistance by using newly developed magnesium alloys, ion implantation may be promising to further improve corrosion resistance and biocompatibility of the substrate.
4. Considering the extremely complex biological environment in human body, composite coatings may be promising with combined properties of different coatings. However, with the increase of interfaces in the composite coatings, the adhesion strength, not only between coating and substrate, but also at interfaces of different layers, has to be paid much attention. A lack of adhesion strength in any interfaces will cause a potential danger of coating dropping.

Considering the properties of magnesium-based materials and the biological environment where they are applied, a proper surface design of magnesium-based implants is critical for their practical applications. The proper surface should have multifunctions to reach the requirement for biomedical applications, so it can be designed as follows:

1. An inner anti-corrosion layer highly adhered to magnesium substrate, such as PEO coating. This layer is aimed to maintain corrosion resistance in long-term service and provide adhesion sites for the following layer.
2. An intermediary layer for further enhanced corrosion resistance and biocompatibility, such as degradable polymers or Ca–P-based coatings. Combined with the inner layer, this layer can further improve the corrosion resistance of magnesium-based implant and meanwhile make up the lack of biocompatibility of inner layer to provide them with better surface biocompatibility for cell attachment and proliferation.
3. An outer layer for biofunctions, such as biofunctional organic molecules. For different biomedical applications, such as orthopedic implants and cardiovascular stents, the requirement for biofunction of the surface is different. For example, in orthopedic applications, the proper surface is required to be suitable for osteoblast cells growth and differentiation while anti-platelet adhesion and fast endothelialization are demanded in cardiovascular stent applications. So grafting different biofunctional organic molecules as outer layer is feasible and flexible to control cells behavior to obtain specific biofunction.

So constructing a proper surface on magnesium-based materials are critical for their usage. To be pointed out, the surface design of magnesium-based materials should base on their application situations, such as implantation sites, surrounding biological environment and required service duration. After proper surface design, magnesium-based materials are considered to be promising as candidate in many biomedical areas.
